# Clear Cell Chondrosarcoma of Bone

**DOI:** 10.1080/13577149977749

**Published:** 1999-09

**Authors:** Khalid S. Ayoub, Robert J. Grimer, Simon R. Carter, David C. Mangham, A. M. Davies, Roger M. Tillman

**Affiliations:** The Royal Orthopaedic Hospital Oncology Service The Royal Orthopaedic Hospital NHS Trust Bristol Road South Northfield Birmingham B31 2AP UK

## Abstract

*Purpose.*  Clear cell chondrosarcoma is a rare variant of chondrosarcoma. Six cases are herein reported.

*Subjects.*  We have treated six patients with clear cell chondrosarcoma in the past 28 years, representing 1.6% of all chondrosarcomas  seen in this time period.

*Results and Discussion.*  Half the patients had been initially underdiagnosed and inappropriately treated.

*Conclusions.*  Our results and our review of the literature highlight the fact that inadequate initial treatment leads to a high rate of both local recurrence and metastasis whilst wide initial excision is usually curative.


Sarcoma (1999) 3, 115–119

ORIGINAL ARTICLE

### Clear cell chondrosarcoma of bone

KHALID S. AYOUB, ROBERT J. GRIMER, SIMON R. CARTER, DAVID C. MANGHAM, A.M. DAVIES & ROGER M. TILLMAN

*The Royal Orthopaedic Hospital Oncology Service, The Royal Orthopaedic Hospital, Birmingham, UK*

**Abstract**
*Purpose*. Clear cell chondrosarcoma is a rare variant of chondrosarcoma. Six cases are herein reported.
*Subjects*. We have treated six patients with clear cell chondrosarcoma in the past 28 years, representing 1.6% of all chondrosarcomas seen in this time period.
*Results and Discussion*. Half the patients had been initially underdiagnosed and inappropriately treated.
*Conclusions*. Our results and our review of the literature highlight the fact that inadequate initial treatment leads to a high rate of both local recurrence and metastasis whilst wide initial excision is usually curative.

**Key words**: *clear cell chondrosarcoma, bone tumour, diagnostic imaging, limb-salvage surgical technique.*

### Introduction

Clear cell chondrosarcoma is a very rare malignant bone tumour. It was initially described by Unni *et al.* in 1976,[1] and was called 'Clear-cell Variant of Chondrosarcoma'. Up to that time, the tumour had usually been mistaken for a benign tumour. Because of it's rarity this lesion is still being confused with benign or more malignant bone tumours, both radiologically and histologically. We have reviewed our experience in treating clear cell chondrosarcoma at the Royal Orthopaedic Hospital, Birmingham, with the advantage of modern methods of diagnostic imaging and limb-salvage surgical techniques.

### Patients and methods

We have undertaken a retrospective review of all patients with clear cell chondrosarcoma treated by the Royal Orthopaedic Hospital Oncology Service, Birmingham, UK. Data has been retrieved from the departmental computerised database supplemented by reviews of the case notes, and histological and radiological studies. Since 1970, six such cases have been treated, representing 1.6% of the 370 cases of chondrosarcoma registered at our centre in the last 28 years.

Five patients were male, and one was female. Their age ranged from 15 to 45 years. The predominant clinical presentation of the tumour was local pain in the affected bone of variable duration. Further clinical details are mentioned in Table 1.

### *Radiographic features*

Initial radiographs were available for review in four of the six patients (Table 1). All the tumours were situated in the epiphyseo-metaphyseal area of the long bones. The most common radiographic features were; an expansile radiolucent bony lesion, absence of any periosteal reaction and absence of soft tissue mass (Fig. 1). Magnetic resonance imaging (MRI) was obtained for two patients. On T1-weighted images, the lesion showed relatively homogeneous low to intermediate signal intensity, and heterogeneous high signal intensity on T2-weighted images (Fig. 2).

### *Pathological features*

On gross histological examination, the tumours showed a lobulated mixed soft and solid lesion, composed of glassy tissue. On microscopic examination, faint microlobular and osteoblastoma-like features predominated on low-power examination (Fig. 3). The constant feature was the presence of large tumour cells, round-to-oval in shape with distinct borders, abundant clear cytoplasm and a centrally located round nucleus (Clear Cell Chondrocytes).[2] Mitotic features were rare, and occasionally these cells showed eosinophilia. The other predominant feature was woven bone trabeculae within the microlobules or scattered between sheets of tumour cells. Multinucleated giant cells were seen in all tumours. There was variable cartilage matrix production.

Correspondence to: Mr R.J. Grimer, Consultant Orthopaedic Oncologist, Royal Orthopaedic Hospital Oncology Service, The Royal Orthopaedic Hospital NHS Trust, Bristol Road South, Northfield, Birmingham B31 2AP, UK. Tel.: 0121 6854150; Fax: 0121 6854146.

1357-714X print/1369-1643 online/99/020115-05 © 1999 Taylor & Francis Ltd

**Table 1**. *Details of six cases of clear cell chondrosarcoma*

| Case | Age/Sex (years) | Clinical presentation | Site | Plain radiographic features | Initial working diagnosis | Treatment | Further treatment | Follow-up | Outcome |
|---|---|---|---|---|---|---|---|---|---|
| 1 | 43/M | Pain for 12 months | Proximal femur | Radiolucent lesion, Thinned cortex | Chondrosarcoma | Wide resection and endoprosthetic replacement | Resection and reconstruction of hemi-pelvis (for local recurrence after 19 years) | 20 years | Alive and free of disease |
| 2 | 27/F | Pain | Ilium | Not available | Chondroblastoma | Curettage | —Curettage (for local recurrence after 34 months) —Curettage (for local recurrence after 4 months) —Hindquarter amputation (for local recurrence after 5 years) | 13 years | Alive and free of disease |
| 3 | 26/M | Pain for 30 monthsProximal femur | Expensile radiolucent lesion, Thinned cortex | Clear cell chondrosarcoma | Wide resection and endoprosthetic replacement | | 2 years | Alive and free of disease | |
| 4 | 15/M | Pain | Distal femur | Not available | Chondromyxoid fibroma | Curettage | Above knee amputation (for local recurrence after 5 years) | 6 years | Died of lung metastasis |
| 5 | 45/M | Asymptomatic, accidental X-ray finding | Distal femur | Expensile radiolucent lesion, Thinned cortex | Clear cell chondrosarcoma | *En-bloc* resection | | 2.5 years | Alive and free of disease |
| 6 | 35/M | Pathological fracture | Proximal femur | Expensile radiolucent lesion, Thinned cortex | Benign tumour/ chondroblastoma | Total hip replacement (Intra-lesional excision) | | 12 months | Alive with disease |

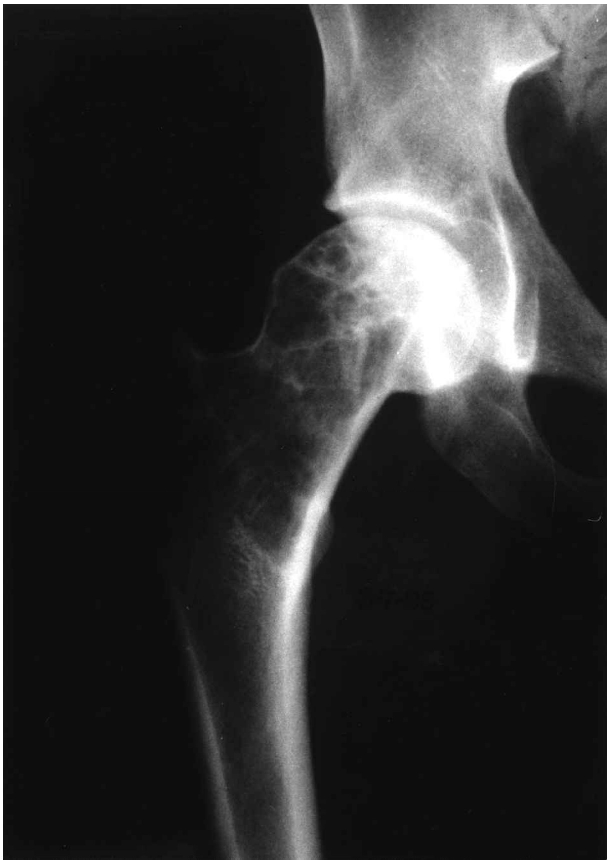

**Fig. 1.** *An anteroposterior view of plain X-ray of the right proximal femur of a 28-year-old man with clear cell chondrosarcoma. It demonstrates an expansile lytic lesion in the femoral neck, with thinning of otherwise intact femoral cortex.*

Immunohistochemical analysis revealed the tumour cells were strongly positive to S-100.

### Treatment and results

The clinical follow-up ranged from 12 months to 20 years (Table 1). Three patients in our study were given 'inappropriate' treatment at the referring institutions due to a mistaken provisional diagnosis of a benign cartilaginous tumour. One of these three patients had curettage of a tumour in her ilium for what was thought a chondroblastoma. She developed local recurrence after 34 months which was treated by further curettage. Within 4 months, she developed a rapid-growing local recurrence of the tumour spreading to the whole hemi-pelvis. Further biopsies then revealed the diagnosis of clear cell chondrosarcoma. The patient was offered radical surgical treatment (amputation) which she declined in order to keep her leg. She underwent a further two-stage curettage of her tumour and bone cement implanted in the defect. Five years later, another recurrence was treated by hindquarter amputation. Eight years after the amputation, she is still alive and free of disease.

The second patient was diagnosed initially as a case of chondromyxoid fibroma of the distal femur which was treated by curettage. After 63 months he

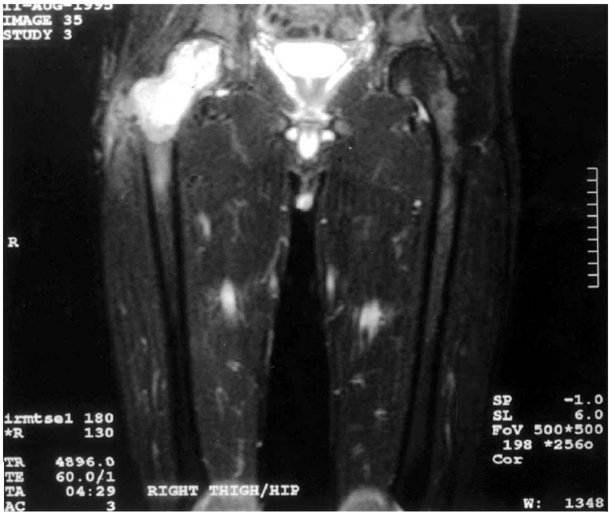

**Fig. 2.** *A coronal view of T2-weighted magnetic resonance imaging (MRI) of pelvis and thighs for the same patient in Fig. 1. It shows the high signal of the tumour in proximal right femur.*

developed a local recurrence treated by above-knee amputation following revision of the diagnosis to clear cell chondrosarcoma. Six months following the amputation he developed lung metastases and died shortly after that.

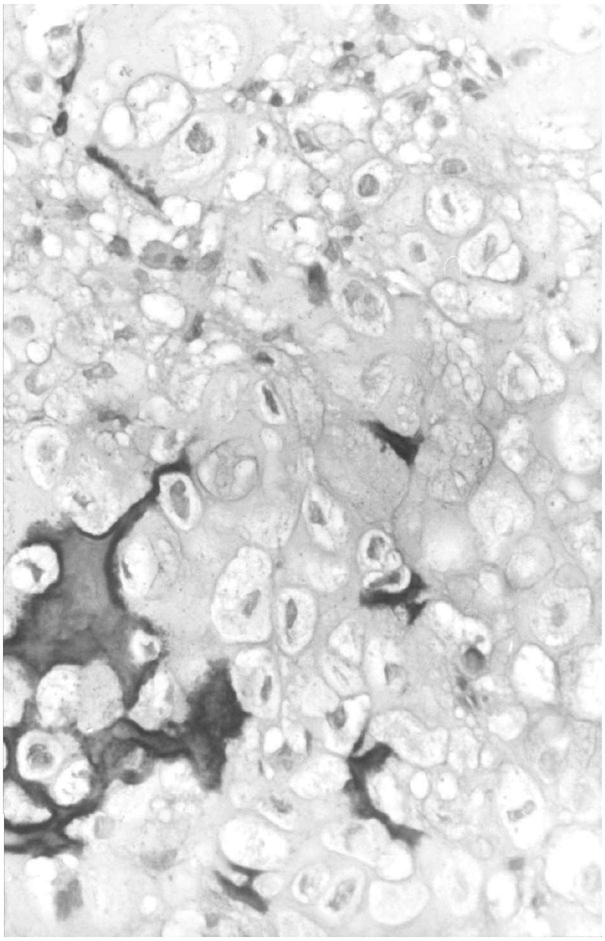

**Fig. 3.** *A high power view of a clear cell chondrosarcoma showing the large tumour chondrocytes, often with clear cytoplasm, and associated partially mineralized hyaline cartilage matrix.*

**Table 2.** *Outcome of treatments by comparison*

| | Bjornosson[3] ($n$=47) | Present[4] ($n$=8) | Laporte[5] ($n$=13) | Our study ($n$=6) | (Total) Average[d] |
|---|---|---|---|---|---|
| I. No of patients with adequate treatment[a] | 22 | 6 | 9 | 3 | (40) |
| Local recurrence | 3(14%) | 0 | 0 | 1(33%) | 10% |
| Metastases | 2(9%) | 0 | 0 | 0 | 5% |
| Deaths | 1(4%) | 0 | 0 | 0 | 2.5% |
| II. No of patients with inadequate treatment[b] | 22 | 2 | 3 | 3 | (30) |
| Local recurrence | 15(68%) | 0 | 2(66%) | 2(66%) | 63% |
| Metastases | 6(27%) | 0 | 1(33%) | 1(33%) | 27% |
| Deaths | 7(32%) | 0 | 1(33%) | 1(33%) | 30% |
| Total no. of patients[c] | 44 | 8 | 12 | 6 | |

[a]\Adequate treatment means complete surgical removal of tumour as primary treatment (i.e. en-bloc resection, amputation).
[b]Inadequate treatment means all treatment methods other than those mentioned in ([a]) above.
[c]No. of patients with only adequate *follow-up information* considered in this table.
[d]The 'Average' is obtained by dividing the sum of patients who had the specific complication (e.g. local recurrence) reported in those series by the total number of patients in either of the main groups (i.e. adequate or inadequate treatment group), multiplied by 100.

The third patient presented to another hospital with a pathological fracture of the neck of femur with a provisional clinico-radiological diagnosis of a benign tumour, possibly chondroblastoma, at the fracture site. He had a total hip replacement at the referring hospital. Histological study of the resected bone confirmed the diagnosis of clear cell chondrosarcoma. At 12 months follow-up (the time of writing this article), there is no evidence of disease.

The other three patients all had the correct initial diagnosis of clear cell chondrosarcoma made on presentation. All had primary wide resection of the tumour with satisfactory outcomes (Table 1). One of these three patients had a primary resection and endoprosthetic replacement of his proximal femur in 1977. In 1996 he had revision surgery for aseptic loosening of the acetabular component of his prosthesis, and the histological study of the 'curettage' taken from the acetabulum showed no malignancy. In early 1997, the patient started to show evidence of local recurrence of the tumour in the ipsilateral ilium. He underwent en-block excision of the ilium with a wide margin and reconstructive procedure for his hemi-pelvis. The histology report of the resected specimen confirmed the diagnosis of clear cell chondrosarcoma, recurring 19 years following the initial treatment. He remains disease-free two years later.

In summary, six patients were included in this study. Three patients had mis-diagnosis of the tumour and treated with intra-lesional procedures. This resulted in two local recurrences, and one death due to lung metastasis. The other three patients in this study had correct diagnosis and appropriate treatment initially for the tumour resulting in good outcomes.

### Discussion

Clear cell chondrosarcoma was described by Unni *et al.* in 1976 when they published a series on 16 patients.[1] It is a very rare malignant bone tumour, comprising 1.6–5.4% of all chondrosarcoma,[3–5] 0.2% of biopsy-analysed primary bone tumours.[2] Various suppositions have been made regarding the source of this tumour. Some considered that the most likely source of this rare chondrosarcoma variant is the same group of cells that give rise to chondroblastoma.[2] Others suggested that it may represent the malignant counterpart of chondroblastoma.[6]

The age at initial presentation ranges from 13 to 85, although most patients will be in their third to fifth decades. There is a male predominance of 1.6–2.6:1.[3,4] Clear cell chondrosarcoma involves the proximal end of the long bone in 75% of the cases (60% femur, 15% humerus), 15% around the knee (distal femur and proximal tibia). In about 10%, the lesion has been noted in the skull, spine, ribs, pelvis, ulna or phalanges.[3] There have been case reports of clear cell chondrosarcoma involving more than one bone.[1] Clinically, the most common presenting symptom is local pain of long duration. Bjornsson *et al.* mentioned in his series of 47 cases, the largest series published, that 55% of the patients had symptoms lasting for more than 1 year before their presentation.[3] Other frequent forms of presentation were swelling, various disabilities affecting the adjacent joint and pathological fractures (from 2%[2] to 37%[1]). Some of the others were asymptomatic and the lesion discovered as an incidental finding on plain x-ray.

Radiographically, when the tumour affects the long bone, the lesion is almost always located in the epihyseal area with or without extension to the metaphysis. Very few cases are reported with tumour extending to or located entirely in the diaphysis.[3,7] The most common radiographic features on plain X-ray, as described in this study and the previous literature,[1–4,7] are; radiolucency (83%), expansion of the bone (75%), densification/calcification (45%), a border between tumour and host bone with or without sclerotic rim (49%), cortical involvement, often in the form of thinning (71%), the cortex was intact in the majority of cases and periosteal reaction (3%). Soft tissue masses are very rare (less than 10%). On computed tomography (CT), the appearances are similar to those seen on plain X-ray but CT is more sensitive at detecting calcification and cortical involvement.[8,9] The magnetic resonance imaging (MRI) of this tumour tends to show a well demarcated geographic lesion with intermediate signal intensity on T1-weighted images, and a higher signal intensity on T2-weighted images.[10–12] All these features are non specific. Differential diagnoses frequently reported include chondroblastoma, giant cell tumour, chondroma, chondromyxoid fibroma and cysts. If the tumour showed any malignant features, the differential diagnoses include osteosarcoma, malignant fibrous histiocytoma and fibrosarcoma.

The slow-growing nature of clear cell chondrosarcoma and the apparently benign radiographic and histological appearances in the majority of cases often results in difficulty in diagnosing the tumour at an early stage, with subsequent provision of conservative treatment. Various treatment modalities were used with fairly consistent outcomes throughout many published series. Intra-lesional surgical removal of the tumour (e.g. curettage, incomplete excision) yields an unacceptably high local recurrence of approximately 83[1] to 86%,[3] and death rate from 29[3] to 50%.[1] In contrast, complete surgical removal of the tumour with a wide margin of disease-free tissue (e.g. en-bloc resection, primary amputation) has given a lower local recurrence rate of less than 15%,[3] and much lower death rate.[1,3,5] Radiotherapy was employed as an initial treatment with some claimed local control but poor outcome.[1,3] Amputation was used in a few patients, usually when the initial diagnosis was incorrect and hence amputation was a salvage procedure.[1,3] From our experience in this study and from the larger published studies,[1,3–5] we confirm that inadequate treatment (e.g. curettage, incomplete excision, radiotherapy and chemotherapy as a primary treatment) certainly yields a poorer result compared with the adequate treatment (en-block excision of the tumour as a primary treatment) summarised in Table 2. Only patients with adequate follow-up information are considered in this table for comparison.

### Conclusion

Clear cell chondrosarcoma is a low-grade and slow-growing tumour. Cure can be achieved by wide local excision in the majority of cases. Awareness of the condition and the diagnostic pitfalls will help improve both detection and survival.

### References

1 Unni KK, Dahlin DC, Beabout JW, Sim FH. Chondrosarcoma: Clear-cell variant. *J Bone Joint Surg [Am]* 1976; 58A: 676–83.
2 Mirra JM. Intramedullary cartilage- and chondroid-producing tumors. In: Mirra JM, ed. *Bone tumors*. Philadelphia: Lea & Febiger, 1989: 535–46.
3 Bjornsson J, Unni KK, Dahlin DC, Beabout JW, Sim FH. Clear cell chondrosarcoma of bone: observations in 47 cases. *Am J Surg Pathol* 1984; 8: 223–30.
4 Present D, Bacchini P, Pignatti G, Picci P, Bertoni F, Champanacci M. Clear cell chondrosarcoma of bone: a report of 8 cases. *Skeletal Radiol* 1991; 20: 187–91.
5 Laporte C, Anract P, Tomeno B, Forest M. Clear cell chondrosarcoma: a report of thirteen cases. *Revue de Chirurgie Orthopedique* 1996; 82: 691–9.
6 Schajowicz F. *Tumors and tumorlike lesions of bone and joint*. New York: Springer-Verlag, 1981.
7 Mulder JD, Kroon HM, Schutte HE, Taconis WK. *Radiologic atlas of bone tumors*. Amsterdam: Elsevier, 1993.
8 Leggon RE Jr, Unni KK, Beabout JW, Sim FH. Clear cell chondrosarcoma. *Orthopaedics* 1990; 13: 593–6.
9 Kumar R, David R, Cierney G. Clear-cell chondrosarcoma. *Radiology* 1985; 154: 45–8.
10 Fobben HS, Dalinka MK, Schiebler ML, Burk DL, Fallon MD, Schmidt RG, Kressel HY. The MRI appearances at 1.5 tesla of cartilaginous tumors involving the epiphysis. *Skeletal Radiol* 1987; 16: 647–51.
11 Cohen EK, Kressel HY, Frank TS, Fallon M, Burk DL, Dalinka MK, Schiebler ML. Hyaline cartilage-origin bone and soft-tissue neoplasms: MR appearance and histologic correlation. *Radiology* 1988; 167: 477–81.
12 Bagley L, Kneeland JB, Dalinka MK, Bullough P, Brooks J. Unusual behavior of clear cell chondrosarcoma. *Skeletal Radiol* 1993; 22: 270–82.
